# Primary uterine osteosarcoma arising in a leiomyoma with rapid local recurrence: A case report

**DOI:** 10.1016/j.gore.2022.101102

**Published:** 2022-11-11

**Authors:** Merima Ruhotina, Joanna Kukla, Annemieke Wilcox, Colleen Murphy, Gulden Menderes

**Affiliations:** a Department of Obstetrics and Gynecology, Bridgeport Hospital/Yale New Haven Health, Bridgeport, CT, USA; b Health First Cancer Institute, Melbourne, FL, USA

**Keywords:** Extraskeletal osteosarcoma, Uterine osteosarcoma, Soft tissue neoplasms

## Abstract

**Background:**

Extraskeletal osteosarcoma is an extremely rare malignant neoplasm. Literature regarding primary osteosarcoma of the uterus is confined to only a small number of case reports**.**

**Case**: A 57-year-old female with a history of uterine fibroids presented to the emergency department with abdominal pain. Imaging was notable for an enlarged uterus with a 15 cm calcified fibroid extending along the posterior uterus. The patient underwent a laparotomy for total hysterectomy and bilateral salpingo-oophorectomy. Pathological evaluation of the specimen yielded mesenchymal proliferation with osteoid formation and tumor cells with densely eosinophilic cytoplasm resembling osteoblasts with a final diagnosis of primary uterine osteosarcoma. Multidisciplinary tumor board recommended against adjuvant treatment, given the lack of evidence for improved outcomes for early-stage uterine sarcomas. The patient was followed up with surveillance visits every-three months, entailing physical examination and computed tomography(CT) scans. Unfortunately, she had locoregional oligometastatic recurrence of her disease at 1-year follow up.

**Conclusion:**

Primary uterine osteosarcoma is an extremely rare and aggressive neoplasm with limited understanding regarding optimal treatment options.

## Highlights

• Primary uterine osteosarcoma is an extremely rare and aggressive neoplasm.
• There is no clear consensus about the optimal systemic treatment.
• Chemotherapy treatment options can be deduced from previous studies of bone and soft tissue sarcomas.

## 1 Introduction

Extraskeletal osteosarcoma (ESOS) is a malignant mesenchymal neoplasm that produces osteoid, bone, or chondroid material without demonstrable attachments to bone or periosteum (Choi et al., 2014). This rare tumor can be diagnosed in the trunk, thigh, upper extremities, and retroperitoneum. ESOS represents approximately 2 % to 5 % of osteosarcomas and less than 1 % of all soft-tissue sarcomas^.^ (Allan and Solle, 1971). Uterine sarcomas are uncommon, accounting for 1–2 % of all uterine neoplasms. Pure heterologous osteosarcomas are a rare type of uterine sarcoma with documentation of disease limited to less than 20 case reports (Lin et al., 2002).

In the published literature composed of small number of case reports, the mean age of diagnosis of primary uterine osteosarcoma was 64 years of age (range 41 to 82) (Hardisson et al., 2001). Table 1 depicts a summary of prior published case reports. The main presenting symptoms included abnormal vaginal bleeding; abdominal pain/fullness caused by a large abdominal mass (Tsukasaki et al., 2016). Prognosis was noted to be poor for patients diagnosed with ESOS with five-year survival rate reported as low as 28 %. There is a high risk for recurrence and a dismal survival rate (Jensen et al., 1998, Nystrom et al., 2013). There is limited data regarding adjuvant therapy after surgical management. In this case report,we present a 57-year-old female who presented with primary uterine osteosarcoma arising in a leiomyoma with rapid locoregional recurrence 1 year after initial diagnosis.

**Table 1:** Previous primary uterine osteosarcoma cases.

Author	Year	Patient age	Presenting symptoms	Surgery	Radiation	Chemotherapy	Recurrence or Metastasis	Outcome
Stier and Lyman et al.	1936	53	Abdominal Pain	Hysterectomy + BSO	–	–	None	Deceased after 2 months
Sheffery et al.	1956	67	Vaginal Bleeding	TAH + BSO	–	–	N/A	N/A
Carleton et al.	1961	82	N/A	TAH + BSO	–	–	Lung metastasis	Deceased after 8 months
Karpas et al.	1964	62	Vaginal bleeding	TAH + BSO	–	–	N/A	N/A
Piscioli et al.	1985	56	Vaginal bleeding	TAH + BSO	+	–	Lung metastasis	Deceased
Crum et al.	1990	41	Vaginal bleeding	TAH + BSO	+	Doxorubicin/ CPA/ Dacarbazine	None	Deceased after 4 months
De Young et al.	1992	63	Vaginal bleeding	TAH + BSO	–	–	None	Deceased after 20 days
Emoto et al.	1994	67	Abdominal Pain	TAH + BSO	–	–	Local recurrence	Deceased after 4 months
Hardisson et al.	2001	41	Vaginal bleeding	TAH + BSO	–	Adriamycin/ ifosfamide	Local recurrence	Alive after 8 months
Kostopoulou et al.	2002	56	Abdominal Pain	TAH + BSO, colon resection	–	CDDP/ epirubicin	Local recurrence	Deceased after 6 months
Wang et al.	2011	53	Vaginal bleeding	RH + OMT	–	CDDP/ epirubicin	None	No evidence of disease after 5 months
Kefeli et al.	2012	53	Vaginal bleeding	TAH + BSO	–	–	N/A	N/A
Powell et al.	2014	60	Vaginal bleeding	TAH + BSO	–	–	Lung metastasis and local recurrence	Deceased after 7 months
Abraham et al.	2014	47	Vaginal bleeding	TAH + BSO	–	Doxorubicin/ ifosfamide	Heart and lung metastasis	Deceased after 6 months
Kitawaki et al.	2016	57	Abdominal Pain	TAH + BSO + Appendectomy	–	Docetaxel/gemcitabine	Lung metastasis and local recurrence	Alive after 13 months
Current Case	2021	57	Abdominal Pain	TAH + BSO	–	Doxorubicin/ifosfamide	Local recurrence	Alive after 15 months

TAH, Total abdominal hysterectomy; RH, radical hysterectomy; BSO, Bilateral salpingo-oophorectomy; OMT, omentectomy; CPA, cyclophosphamide; CDDP, cisplatin.

## 2 Case

**Fig. 1:**
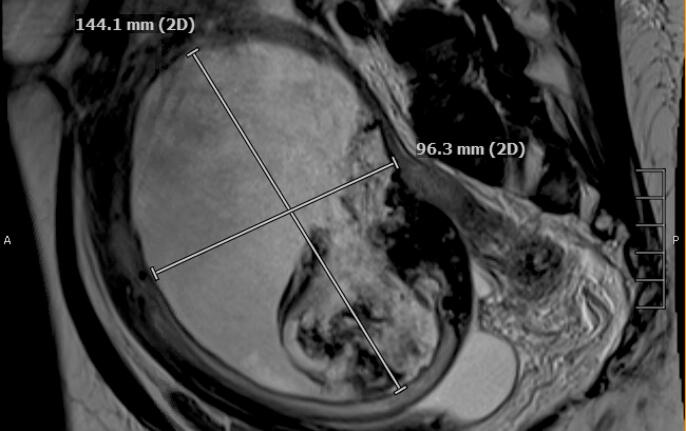
Findings of magnetic resonance imaging. A complex proteinaceous and calcified lesion is visible.

A 57-year-old female with a history of uterine fibroids presented to the emergency department with abdominal pain. Her past medical history was unremarkable. The patient reported intense infraumbilical abdominal pain, abdominal distention, urinary frequency, and incomplete bladder emptying. Her physical exam was notable for large uterus measuring approximately 18 weeks with fullness palpated in the posterior cul-de-sac. Upon admission, she was found to have acute kidney injury with creatinine of 2.04 mg/dL (normal value 0.7 to 1.3 mg/dL). Other laboratory findings including serum electrolytes and hepatic functions were within normal limits. CT of the abdomen and pelvis noted a 15 cm heterogeneous pelvic mass with peripheral coarse calcification, inseparable from the posterior uterine wall and bilateral mild hydroureteronephrosis secondary to mass effect on the distal ureters. A pelvic magnetic resonance imaging (MRI) was notable for an enlarged uterus measuring approximately 19 × 12 × 14 cm with a complex proteinaceous and calcified lesion measuring 15 × 10 × 12 cm extending between the posterior uterine fundus to the lower uterine segment (Fig. 1). No malignant cells were seen on cervical cytology obtained during hospitalization. An attempt was made to perform an endometrial biopsy, however given the position of the cervix, this was not feasible and safe. After discussing the alternatives, the patient agreed to proceed with a total abdominal hysterectomy and bilateral salpingo-oophorectomy. Intraoperative findings included an enlarged fibroid uterus measuring approximately 24 weeks with a large calcified mass measuring 10 × 10 cm, adherent to posterior uterine wall and anterior to the rectosigmoid colon. The remainder of the abdominal survey did not yield any significant findings suggestive of malignancy. An intraoperative frozen specimen pathological evaluation revealed acute inflammation. The patient had an uncomplicated post-operative course and was discharged home on postoperative day 3. Her acute kidney injury was resolved after the surgery, once the compression on distal ureters was relieved with removal of the hysterectomy specimen.

**Fig. 2:**
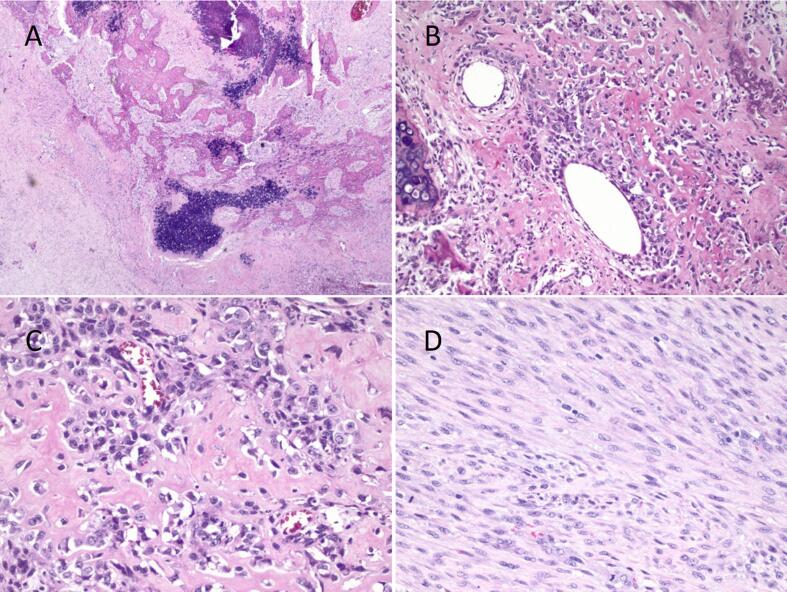
**A-D.** Histological Features of the Uterine Osteosarcoma. Low magnification shows mesenchymal proliferation with osteoid formation (A, HE, 20 x). The tumor cells have densely eosinophilic cytoplasm resembling osteoblasts (B, HE, 100 x) and show significant cytological atypia (C, HE, 200x). The associated leiomyoma consists of proliferation of smooth muscle cells without cytological atypia (D, HE, 100x).

**Fig. 3:**
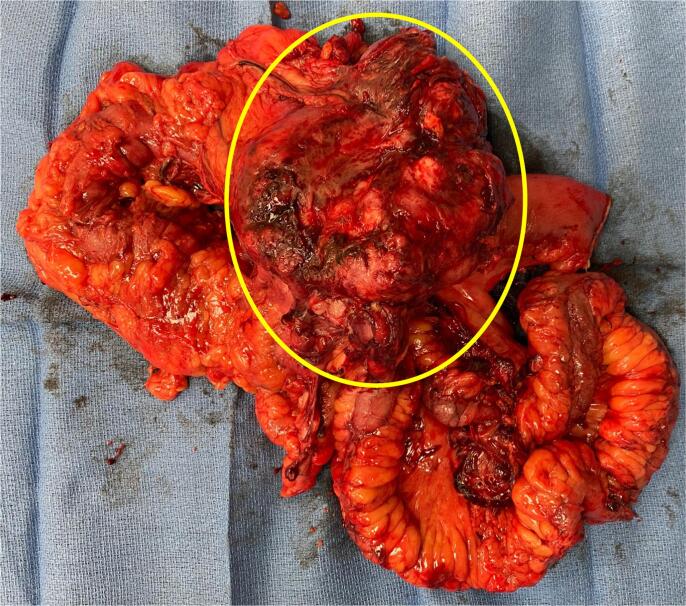
Operative specimen of recurrent mass. Specimen included 15 cm mass in the right pericolic gutter involving the ascending colon from cecum to the hepatic flexure. Mass is encircled.

Final pathological evaluation of the hysterectomy specimen yielded a weight of 701 g and intermediate-grade primary uterine osteosarcoma, measuring 7 × 4 cm arising in a leiomyoma with degenerative changes. The specimen was noted to have dusty red brown, edematous tissue. Microscopically, low magnification showed mesenchymal proliferation with osteoid formation, with tumor cells having densely eosinophilic cytoplasm resembling osteoblasts. There was significant cytological atypia and the associated leiomyoma consisted of proliferated smooth muscle cells without cytological atypia. There was a moderately pleomorphic sarcomatous component, forming thin trabeculae of bone (Fig. 2). A post-operative PET/CT was without evidence of FDG avid distant metastasis. The patient’s case was reviewed at the gynecologic oncology and sarcoma tumor boards with a consensus to proceed with observation. This conclusion was made given the early stage of disease and extrapolation of data from early-stage uterine sarcomas which demonstrated no improvement in oncological outcomes with addition of adjuvant therapy. The patient underwent surveillance with CT chest/abdomen/pelvis and physical exams every 3 months. Approximately 1 year after her diagnosis, she presented to the emergency room with nausea, vomiting, and abdominal pain. A CT of the abdomen and pelvis noted a large 12 cm metastatic peritoneal mass in the right lower abdomen demonstrating invasion of the distal ileal loops and cecum, with resultant proximal small bowel obstruction. After extensive discussion of limited management options, she opted for surgical management and underwent a secondary tumor debulking, which entailed a right hemicolectomy. Intraoperative findings were notable for a 15 × 15 cm mass in the right pericolic gutter involving the ascending colon from cecum to the hepatic flexure (Fig. 3). This large mass was deemed to be the cause of her small bowel obstruction. The patient had an uneventful post-operative course. She has been dispositioned to systemic chemotherapy in the form of doxorubicin and ifosfamide, after recovering from the surgery. The patient has completed two cycles of chemotherapy and she remains alive with cancer.

## 3 Discussion

Extraskeletal osteosarcoma, also known as soft tissue osteosarcoma, is a rare malignant neoplasm that produces osteoid, bone, or chondroid material but lacks bone or periosteum involvement. It is an extremely aggressive tumor with a poor prognosis. The exact etiology of ESOS is still unknown but several associated prognostic factors have been proposed, such as the history of trauma, local radiation therapy, and changes in soft tissue lesions and malignant fibrous tissue disease (Liao et al., 2019). This case report presents a patient with initially confined uterine osteosarcoma and rapid recurrence of disease one year after diagnosis. In this particular case, histological evaluation yielded an absence of an epithelial component, no evidence of osteosarcoma origin in bone, and had presence of neoplastic osteoid. Immunohistochemical staining for smooth muscle cell markers was negative, indicating the absence of leiomyosarcoma components. Given all of these elements, the neoplasm reported fulfills the diagnosis of primary uterine osteosarcoma. As our patient’s osteosarcoma was uterine-confined after primary surgical resection, management was extrapolated from studies of leiomyosarcoma where observation is the standard of care (Byar et al., 2022). A systemic review addressing the effect of adjuvant chemotherapy or radiation on localized ESOS also found no difference in 5-year disease free survival rate between surgery and adjuvant chemotherapy versus surgery alone groups (Tsukamoto et al., 2022). After the patient’s locoregional recurrence was managed surgically, she was initiated on systemic chemotherapy. There is no clear consensus about the optimal chemotherapy regimen for patients with ESOS. For advanced or metastatic leiomyosarcoma cases, the use of gemcitabine plus docetaxel, doxorubicin (with or without ifosfamide), single-agent gemcitabine, ifosfamide, trabectedin, pazopanib, and dacarbazine are recommended (NCCN, 2020). The standard of care for high grade bone sarcomas includes a multi-agent regimen with a combination of doxorubicin, cisplatin +/− methotrexate or ifosfamide (Ferrari et al., 2018). There are some case reports suggesting the use of gemcitabine-docetaxol as a potential regimen for long term progression free survival (Strippoli et al., 2015). For our current case report, treatment options were deduced from previous studies of bone and soft tissue sarcomas. The patient is currently on her second cycle of chemotherapy and tolerating treatment well.

In summary, we present a 57-year-old female with a rare diagnosis of primary uterine osteosarcoma. Most notably in this case was the rapid and aggressive locoregional recurrence, approximately 1 year after initial diagnosis. This case adds to the growing but still sparce literature surrounding this rare tumor. There are limited data regarding systemic treatment options. Therefore, until more effective systemic treatment options are developed, surgical resection remains the primary treatment in the up-front and recurrence settings.

Written informed consent and IRB review was obtained from the patient for publication of this case report and accompanying images. A copy of the written consent is available for review by the Editor-in-Chief of this journal on request.

## CRediT authorship contribution statement

**Merima Ruhotina:** Conceptualization, Writing – original draft. **Joanna Kukla:** Investigation, Writing – original draft. **Annemieke Wilcox:** Investigation, Writing – review & editing. **Colleen Murphy:** Investigation. **Gulden Menderes:** Conceptualization, Writing – review & editing, Supervision.

## Declaration of Competing Interest

The authors declare that they have no known competing financial interests or personal relationships that could have appeared to influence the work reported in this paper.

## References

[b0005] Allan C.J., Solle E.H. (1971). Osteogenic sarcoma of the somatic soft tissues.Clinicopathologic study of 26 cases and review of literature. Cancer.

[b0010] L. Byar, MSN, APN, BC, BMTCN K, Fredericks, MD, MPH T. Uterine Leiomyosarcoma. J. Adv. Practition. Oncol. 2022 Jan 1;13(1):70–6. 10.6004/jadpro.2022.13.1.6 PMC8805803 35173990

[b0015] Choi L.E., Healey J.H., Kuk D., Brennan M.F. (2014). Analysis of Outcomes in Extraskeletal Osteosarcoma. J. Bone Joint Surg..

[b0020] Ferrari S., Bielack S.S., Smeland S., Longhi A., Egerer G., Sundby Hall K. (2018). EURO-B.O.S.S.: A European study on chemotherapy in bone-sarcoma patients aged over 40: Outcome in primary high-grade osteosarcoma. Tumori J..

[b0025] Hardisson D., Simón R.S., Burgos E. (2001). Primary Osteosarcoma of the Uterine Corpus: Report of a Case with Immunohistochemical and Ultrastructural Study. Gynecol. Oncol..

[b0030] Jensen M.L., Schumacher B., Jensen O.M., Nielsen O.S., Keller J. (1998). Extraskeletal Osteosarcomas. Am. J. Surg. Pathol..

[b0035] Liao Z., Qiu M., Yang J., Yang Y., Zhu L., Yang B. (2019). Outcomes of surgery and/or combination chemotherapy for extraskeletal osteosarcoma: a single-center retrospective study from China. Sci. Rep..

[b0040] Lin J.-W., Ko S.-F., Ng S.-H., Eng H.-L., Changchien C.-C., Huang C.-C. (2002). Primary osteosarcoma of the uterus with peritoneal osteosarcomatosis: CT features. Brit. J. Radiol..

[b0045] National Comprehensive Cancer Network, 2020. NCCN Clinical Practice Guidelines in Oncology: Uterine neoplasms.

[b0050] Nystrom L.M., Reimer N.B., Reith J.D., Dang L., Zlotecki R.A., Scarborough M.T. (2013). Multidisciplinary Management of Soft Tissue Sarcoma. Scient. World J..

[b0055] Strippoli S., Traversa M., Cramarossa A.A., Popescu O., Lorusso V., Guida M. (2015). Long-term response of gemcitabine plus docetaxel chemotherapy regimen for extraskeletal osteosarcoma: A case report. Oncol. Lett..

[b0060] Tsukamoto S., Mavrogenis A.F., Angelelli L., Righi A., Filardo G., Kido A. (2022). The Effect of Adjuvant Chemotherapy on Localized Extraskeletal Osteosarcoma: A Systematic Review. Cancers..

[b0065] Tsukasaki N., Mori T., Yasukawa S., Konishi E., Kokabu T., Kitawaki J. (2016). Primary osteosarcoma of the uterine corpus: A case report. J. Obstetr. Gynaecol. Res..

