# Supplementary material for: Primary uterine osteosarcoma arising in a leiomyoma with rapid local recurrence: A case report
Source: Gynecol Oncol Rep. 2022 Nov 11;44:101102. doi: 10.1016/j.gore.2022.101102 (PMC9672400; doi:10.1016/j.gore.2022.101102)
Supplement: Supplementary data 1 [file mmc1.pdf]

**Yale University**

Human Investigation Committee
Human Subjects Committee
25 Science Park – 3rd Fl., 150 Munson St.
New Haven CT 06520-8327

Telephone: 203-785-4688
http://www.yale.edu/hrpp

# "NOT RESEARCH" DETERMINATION

9/26/2022

Merima Ruhotina
merima.ruhotina@gmail.com

Dear Merima Ruhotina:

On 09/26/2022, the Yale IRB reviewed the following submission:

| | |
|---|---|
| Type of Review: | Initial Study |
| Title of Study: | Primary uterine osteosarcoma arising in a leiomyoma with rapid local recurrence: a case report |
| Investigator: | Merima Ruhotina |
| Documents: | • A case of primary uterine osteosarcoma |

The Yale IRB determined that the proposed activity does not constitute "research" as defined in 45 CFR 46. Note that this regulation defines research as "a systematic investigation, including research development, testing and evaluation, designed to contribute to generalizable knowledge."

The description of your project as you have submitted it to the IRB does not meet the regulatory definition of research and as such, does not require IRB review or approval. This determination applies only to the activities described in the IRB submission and does not apply should any changes be made. If changes are being considered and there are questions about whether IRB review is needed, please contact the IRB Office.

Sincerely,

Human Investigation Committee
Human Subjects Committee
