# Supplementary material for: Primary uterine osteosarcoma arising in a leiomyoma with rapid local recurrence: A case report
Source: Gynecol Oncol Rep. 2022 Nov 11;44:101102. doi: 10.1016/j.gore.2022.101102 (PMC9672400; doi:10.1016/j.gore.2022.101102)
Supplement: Supplementary data 2 [file mmc2.pdf]

NAME: McFarlane, Marjorie Angela
MRN: MR6786372 CSN:291900151
DOB: 11/3/1964 (57 yrs) Sex: F 203-570-3671
BIRTH DATE: Date of Service: 7/8/2022
Prov: 26773 [Clark, Mitchell Brent,
MRN: 291900151
DOS:

MARJORIE ANGELA

(If handwritten, patient name, MRN, birth date, and DOS)

# YALE NEW HAVEN HEALTH

## Consent for Operation or Special Procedure

**This form is available in multiple languages. Please use an interpreter and the appropriate consent form for patients who do not speak English.**

**SECTION A:**

1. After discussing other options, including no treatment, with the responsible practitioner or his/her delegated representative, I give *(insert name of person performing procedure)* Dr. Mitchell Clark permission to perform the following operation, procedure(s) or treatment *(list name or description of operation(s), procedure(s) and/or treatment(s) - indicate applicable level, side, or site)*:

   Exploratory laparotomy, hemicolectomy, tumor debulking, possible ostomy, and other indicated procedures

   I understand that this procedure is for purposes of diagnosis and/or treatment for *(describe reasons for procedure)*:

   uterine osteosarcoma, pelvic mass

2. I give permission to my responsible practitioner to do whatever may be necessary if there is a complication or unforeseen condition during my procedure.

3. My responsible practitioner has explained to me in a way that I understand: (a) the nature and purpose of the procedure(s); (b) the potential benefits and risks and possible side effects of the procedure(s) both during it and during recuperation, including bleeding, infection, accidental injury of other body parts, failure to permanently improve my condition or death, as well as the potential risks and benefits of the medications that may be administered to me as part of the procedure; and (c) the alternative(s) to the procedure(s) and their potential risks and benefits, including the option of not having the procedure. I understand that other complications may occur, including but not limited to:

   Bleeding, infection, injury to surrounding structures, needing further procedures

   ☐ **(Contents of discussion including risks, benefits and alternatives are documented in an office or hospital chart note)**

4. I understand the purpose and potential benefits of the procedure in relation to my goals. My responsible practitioner has explained to me what results to expect, and the chances of achieving them. I understand that no promises or guarantees have been made or can be made about the results of the procedure(s).

5. I agree to have anesthesia as necessary to perform the procedure(s). I understand that if an anesthesiologist is to be involved he/she will speak to me about the risks of anesthesia in more detail and I may be asked to sign a separate anesthesia or sedation consent form.

6. I give permission to the hospital and/or its departments to examine and keep tissue, blood, body parts, or fluids removed from my body during the procedure(s) to aid in diagnosis and treatment, after which they may be used for scientific research or teaching by appropriate persons. If these things are used for science or teaching, my identity will not be disclosed. I will no longer own or have any rights to these things regardless of how they may be used.

7. If the procedure listed above involves the implantation/transplantation of tissue from a human or animal source, my responsible practitioner has described to me the risks and benefits of, and alternatives to, receiving this product.

8. I understand that some of the system hospitals are teaching hospitals. Doctors or other health practitioners who are members of the care team and are in training may help my practitioner with the procedure. I understand that these trainees are supervised by qualified staff and the responsible practitioner will be present at all important times during the procedure. I also understand that associate(s), surgical assistants and/or other non-physicians or trainees may assist my responsible practitioner or perform parts of the procedure under the responsible practitioner's supervision, as permitted by law and hospital policy. This includes compliance with the overlapping surgery policy which ensures that the attending surgeon will be present for the critical and key portions of my case and that an alternate attending physician will be designated should the need arise. If others who are not hospital staff will be present in the operating room, the responsible practitioner has spoken with me about this. I understand that a representative of an equipment vendor or a visitor may be present in the procedure area and that if that occurs, any visitor or vendor will comply with any applicable policy regarding observers in the Operating Room or other procedural area.

9. I give permission to the hospital and the above-named practitioner to photograph and/or visually record or display the procedure(s) for medical, scientific, or educational purposes. I understand that I will not be identified to those not involved in my care unless a separate consent is signed.

[1] In cases of refusal of blood by a parent or guardian of a minor in a situation in which transfusion may be anticipated, contact Legal and Risk Services immediately, as in most cases court intervention will be sought

NAME: McFarlane, Marjorie Angela MRN: MR0788372 CSN:291900151 ...RIE ANGELA
BIRTH DATE: DOB: 11/3/1964 (57 yrs) Sex: F 203-570-367
MRN: Date of Service: 7/8/2022 Prov: 26773 [Clark, Mitchell Brent,
291900151
DOS:

(If handwritten, patient name, MRN, birth date, and DOS)

**YALE NEW HAVEN HEALTH**

**Consent for Operation or Special Procedure**

10. I understand that my responsible practitioner may deem it necessary for me to have a blood transfusion during or after the procedure(s). I understand what a blood transfusion is, the procedures used, the benefits of receiving a transfusion and the risks involved. The benefits include better oxygen delivery to all parts of my body (for red blood cells) and treating or decreasing the risks of bleeding (for platelets and plasma products). The risks include: fever, chills, and allergic reactions which are generally mild and transient; on rare occasions major transfusion reactions occur such as rapid breakdown of blood cells and acute lung or kidney injury; and rarely bacterial, viral or other infections such as hepatitis B, hepatitis C, human immunodeficiency virus (HIV) and other pathogens. I understand these risks exist, although screening and testing of blood donors and their blood is performed to minimize these risks. My questions regarding alternatives have been addressed by the responsible practitioner in relation to my specific circumstances.

| | | |
|---|---|---|
| MM (Initial) I **agree** to receive transfusions of blood or blood products if medically necessary. | **OR** | ____ (Initial) I **decline** to receive any transfusions of blood or blood products and understand that I may suffer severe injury or death as a result of my refusal.[1] |

***(Patient INITIAL one or the other)***

11. In the event a healthcare worker is exposed to my blood or body fluids in connection with my procedure, or during my hospital stay, I agree to the collection and testing of my blood for HIV.

12. I have read this form or had it read to me. I have had an opportunity to ask questions and to consider my decisions. All of my questions have been answered to my satisfaction.

| Time | Date | Signature | Printed Name |
|---|---|---|---|
| 1300 | 7/11/22 | [signature] (Signature of Person Obtaining Consent) | Yifan Chang (Printed Name of Person Obtaining Consent) |
| 1300 | 7/11/22 | x Marjorie McFarlane (Patient Signature) | x Marjorie McFarlane (Patient Printed Name) |
| | | (Signature of Authorized Representative (person consenting for patient)) | (Printed Name of Authorized Representative) |

Relationship to Patient ____ ☐ patient too severely ill ☐ patient unconscious ☐ patient lacks capacity ☐ patient is a minor

Name/code of the interpreter: ____ Company if other than hospital: ____

☐ Interpreter info. recorded elsewhere in office or hospital chart

**SECTION B – TELEPHONE CONSENT:**

I have discussed in a witnessed telephone conversation all of the issues set forth in the **CONSENT FOR OPERATION OR SPECIAL PROCEDURE** with the patient's authorized representative. This included a discussion of the risks, their likelihood, and alternative treatment options as set forth in Section A, above.

**Consent was obtained by telephone on:** __/__/__ (Date) ____ (Time) AM/PM

**Name of person who gave consent:** ____

Relationship to Patient: ____

Time ____ Date ____ Signature of Person Obtaining Consent ____ Printed Name of Person Obtaining Consent ____

Time ____ Date ____ Signature of Witness ____ Printed Name of Witness ____

Name/code of the interpreter: ____ Company if other than hospital: ____

☐ Interpreter info. recorded elsewhere in office or hospital chart

**SECTION C – EMERGENCY PROCEDURE:**

The patient is in need of a procedure to save the patient's life, limb or organ and is unable to consent for him/herself and family is currently unavailable despite reasonable efforts.

Time ____ Date ____ Signature of Responsible Practitioner ____ Printed Name of Responsible Practitioner ____

**SECTION D - Signature of Responsible Practitioner if Other than Person Obtaining Consent in Previous Sections**

Time ____ Date ____ Signature of Responsible Practitioner ____ Printed Name of Responsible Practitioner ____



Generated On: 09/26/2022 15:09
